# Case Report: Pediatric osteogenesis imperfecta type 1: a pedigree report and literature review

**DOI:** 10.3389/fped.2026.1944269

**Published:** 2026-10-21

**Authors:** Rong Rong, Xinyu Li, Xuhan Liu

**Affiliations:** Department of Endocrinology, Dalian Municipal Central Hospital Affiliated to Dalian University of Technology, Dalian, China

**Keywords:** case report, *COL1A1* gene, gene variant, literature review, osteogenesis imperfecta

## Abstract

Osteogenesis imperfecta (OI), also known as “brittle bone disease,” is a congenital disorder typically inherited in an autosomal dominant manner. It is a rare genetic disease caused by abnormal mesodermal development leading to defective collagen formation due to variants in various genes. OI is a hereditary connective tissue disorder characterized by increased bone fragility and recurrent fractures. It can also involve the eyes, ears, teeth, and skin, manifesting as bone deformities, blue sclerae, short stature, dentinogenesis imperfecta, hearing impairment, and laxity of skin, tendons, and ligaments. Its pathogenesis is closely linked to variants in collagen-encoding genes or related regulatory genes. This article reports a case of a 13-year-old male with OI, whose chief complaint was “congenital blue sclerae and recurrent fractures from early childhood”. Based on his family history, clinical examination, and genetic testing results, a definitive diagnosis of OI type 1 was established. This paper further explores the clinical characteristics, diagnostic key points, treatment progress, gene variant correlations, and management considerations of OI, aiming to provide more comprehensive references for clinical diagnosis and treatment.

## 1 Introduction

OI is a group of hereditary connective tissue disorders characterized by the core feature of increased bone fragility, with an incidence of approximately 1/10,000 to 1/20,000, showing no gender predominance (1). The etiology primarily involves abnormalities in collagen synthesis or structure. Among these, the *COL1A1* (HGNC:2197) and *COL1A2* (HGNC:2198) genes, encoding the *α*1 and *α*2 chains of type I collagen respectively, are the most common causative genes, with variants in these two genes accounting for about 90% of OI patients (2). Specifically, *COL1A1* variants are associated with blue sclerae and dental abnormalities, while *COL1A2* variants are closely linked to reduced bone mineral density(BMD). Based on clinical phenotypes and genotypes, OI can be classified into multiple subtypes. With advancements in molecular genetics, the classification framework for OI has expanded substantially. The classic 1979 Sillence classification represents a phenotype-driven clinical system defining four major OI subtypes (types I-IV) according to clinical severity and radiographic features (1, 3). Subsequent molecular discoveries have identified many additional disease-causing genes, giving rise to over 21 genetically-defined OI-related entities that integrate molecular-genetic criteria alongside clinical observations (1, 3). Van Dijk and Sillence later proposed a revised nosology, highlighting the conceptual distinction between clinical severity grading and gene-based numerical subtyping, and recommending Arabic rather than Roman numerals for phenotypic designation (4). Beyond the classic collagen-related causative genes, newly-identified recessive pathogenic variants in genes including *TENT5A, MESD, KDELR2,* and *CCDC134* have been reported in patients with OI-like phenotypes. These variants disrupt LRP5/6 and MAPK/ERK signaling pathways and broaden our understanding of OI-associated molecular mechanisms. Under current classification frameworks, these genes are not designated distinct numeric OI subtypes (5).

The core clinical features of various OI types are as follows (1, 6):

- **Type 1:** The mildest clinical form, caused by insufficient quantity of type I collagen. It is commonly characterized by low bone mass, blue sclerae, and hearing loss. Significant skeletal deformities and growth retardation are usually absent; dental abnormalities are less common.
- **Type 2:** Perinatally lethal form. Severe long bone deformities, multiple rib fractures, and poor skull ossification can be detected prenatally. Sclerae are often dark blue to blue-gray.
- **Type 3:** The most severe type among surviving patients, classified as a progressively deforming type. Bone fragility is very high, predisposing to multiple fractures and progressive skeletal deformities. Some patients may have hearing impairment, dental abnormalities, and blue sclerae.
- **Type 4:** The severity lies between types 1 and 3, primarily characterized by mild to moderate skeletal deformities; hearing impairment is relatively rare.
- **Other Subtypes:** Besides the classic subtypes, the clinical manifestations of newly identified autosomal recessive and X-linked subtypes are heterogeneous. Some may present with specific complications, necessitating genetic testing for definitive diagnosis.

Type 1 is the most common subtype, accounting for 50%–60% of all OI cases. It follows autosomal dominant inheritance and has relatively mild clinical symptoms, mainly presenting as blue sclerae, mildly to moderately reduced BMD, and recurrent fractures after minor trauma. Significant skeletal deformities are typically absent, and the incidence of hearing loss is low, leading to a relatively favorable prognosis (2).

The diagnosis of OI relies on a comprehensive approach combining phenotypic assessment, clinical manifestations, family history, imaging findings, and molecular genetic testing (7). Specifically, phenotypic diagnosis is based on typical clinical features, including blue sclerae, recurrent pathological fractures, low BMD, skeletal deformities, hearing impairment, dental abnormalities, and familial inheritance (often autosomal-dominant, with occasional autosomal-recessive forms or sporadic de-novo-occurring cases) (1, 3). In contrast, molecular diagnosis is defined by the detection of pathogenic variants in collagen-related genes, particularly *COL1A1* and *COL1A2,* which provides definitive molecular evidence for diagnosis and accurate subtyping (1, 3). Additionally, imaging studies can further support the diagnosis by revealing osteoporosis, fractures, vertebral compression deformities, and other skeletal abnormalities. Collectively, the integration of phenotypic evaluation and molecular genetic testing is essential for achieving a clear, accurate, and definitive diagnosis of OI (7).

### Scope of the narrative literature review

As reflected in the manuscript title, this work is a multigenerational pedigree case report accompanied by a narrative literature review. The narrative review focuses on three domains: (1) the clinical classification and phenotypic spectrum of osteogenesis imperfecta; (2) the genotype-phenotype correlation of *COL1A1* variants in OI 1; (3) current therapeutic strategies for paediatric osteogenesis imperfecta. This is not a formal systematic review following PRISMA guidelines; published studies were narratively synthesised to contextualise the clinical, genetic and therapeutic findings observed in our pedigree case.

## 2 Case description

### 2.1 General information

The patient, a 13-year-old male, was admitted to our hospital in 2024. He presented with congenital blue sclerae and a history of multiple low-trauma fractures starting in early childhood. He had not received systematic evaluation previously. Following persistent joint pain after multiple conservatively-managed fractures, he was referred to the Endocrinology Department to investigate the cause of osteoporosis and bone fragility, screen for inherited bone disorders, and establish a long-term bone-health management plan.

### 2.2 History of present illness

The patient has congenital blue sclerae and sustained multiple fractures triggered by minor trauma or falls. At age 3, he fell from playground equipment in kindergarten, resulting in fractures of the right shoulder and clavicle. Between June 2019 and 2024, he sustained nine additional fractures from incidents such as collisions with others or objects, falls, and landings after exercise. These included fractures of the left second metacarpal, right distal radius (twice), right distal ulna (twice), left clavicle, left distal radius, right distal radius, and left knee joint (twice). All fractures were managed conservatively, with follow-up indicating good healing. At age 8, he was diagnosed with “flat feet” at Dalian Children's Hospital. Throughout the disease course, there has been no significant hearing impairment, recurrent dental caries or tooth looseness, vision loss, or symptoms like chest tightness or shortness of breath. His appetite and sleep were normal, with no significant recent weight change.

### 2.3 Past medical history, birth history, and developmental history

He was previously healthy with no other chronic diseases. He is the first child, born at term via cesarean section due to malpresentation, with no history of birth asphyxia. His mother was healthy during pregnancy. He was breastfed until 12 months of age, with complementary foods introduced at 6 months. Vitamin D and calcium supplementation began at 1 month of age and has been taken intermittently since. Developmental milestones were normal: he smiled, lifted his head, and rolled over by 3 months; sat independently and had tooth eruption by 6 months; spoke by 12 months; and walked by 14 months.

### 2.4 Family history

The family shows a clear autosomal dominant inheritance pattern. His mother, maternal grandmother, maternal great-grandfather, maternal great-aunt, maternal cousin (female), and maternal older cousin (female) all have a history of blue sclerae and fractures.

### 2.5 Admission physical examination

Relevant clinical photographs are presented in Figures 1A–C (Table 1).

**Figure 1:**
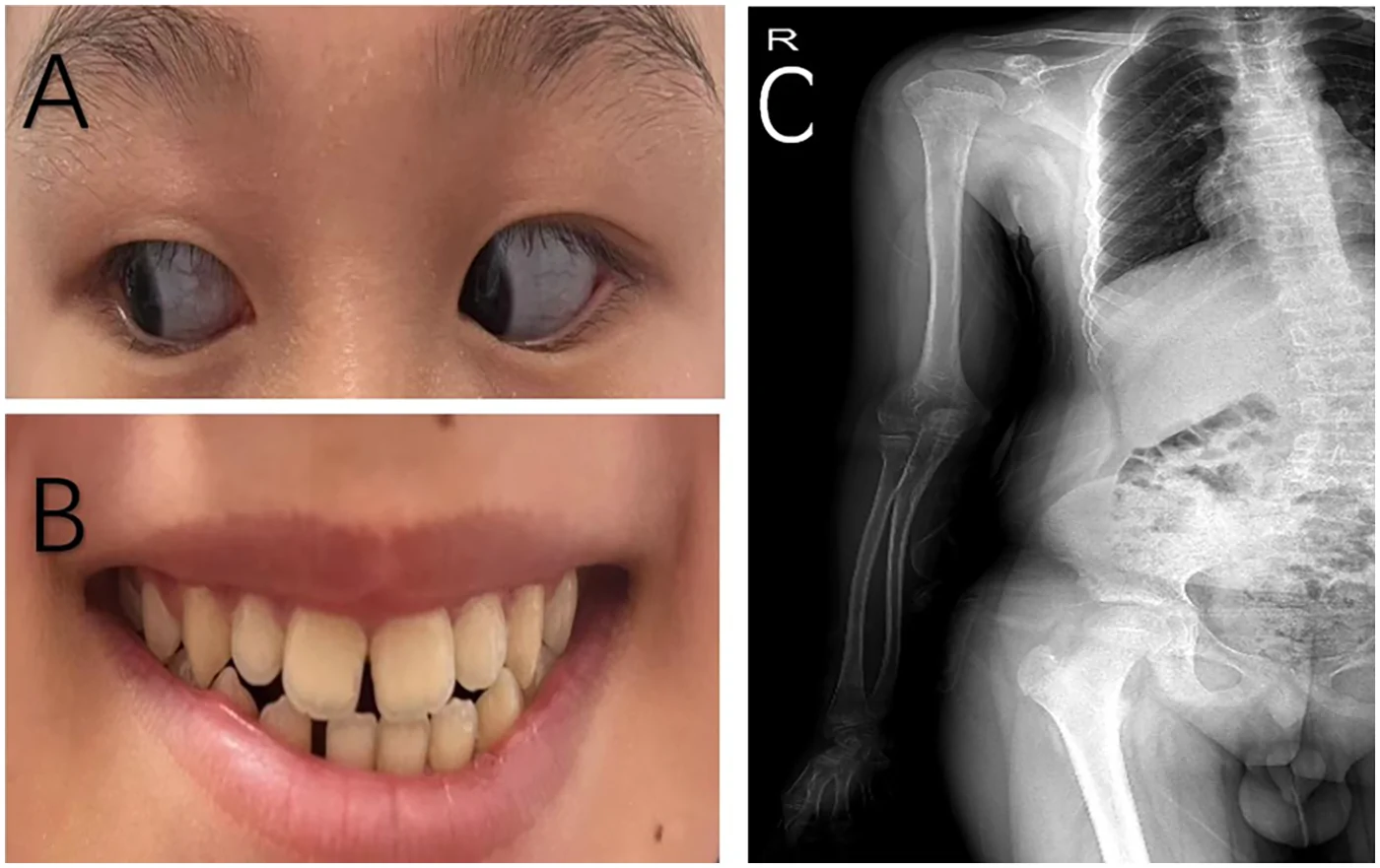
Clinical photographs of the patient. **(A)** Blue sclerae.**(B)** Dental alignment. **(C)** x-ray of mild left-sided spinal curvature.

**Table 1:** Admission physical examination.

Characteristic	Finding	Approx. Z-score/Percentile
Temperature	36.2 °C	—
Pulse	88 beats/min	—
Respiratory rate	20 breaths/min	—
Blood pressure	107/77 mmHg	—
Weight	55 kg	+1.18/88th
Height	159 cm	−0.14/44th
Body mass index	21.8 kg/m^2^	+1.20/88–90th
Arm span	150 cm	NA
Upper segment	79 cm	NA
Lower segment	80 cm	NA
Secondary sexual characteristics	No beard, axillary hair, or pubic hair	—
Intelligence and olfaction	Normal; no red-green color blindness	—
Breasts	Bilaterally slight prominent and soft; no palpable glandular tissue beneath the nipple-areola complex; breast buds absent (lipomastia)	—
Testes	Testes: Soft, non-tender; testicular volume ：Left: 4 mL, Right: 6mL	—
Penile length	7.0 cm	—
External genitalia	Tanner stage III	—
Sclerae	Blue; no jaundice	—
Cardiopulmonary and abdomen	Unremarkable	—
Extremities	No deformities; full range of motion; no tenderness	—
Spine	Mild left-sided scoliosis	—

Z-score and percentile values for height and BMI are approximate, calculated from Chinese national growth standards WS/T 612-2018 and WS/T 586-2018 for 13-year-old boys. NA indicates no available national reference data for these anthropometric indices in Chinese adolescents; (—) denotes parameters without applicable anthropometric standard scores.

Pedigree of the family with osteogenesis imperfecta type 1, drawn in accordance with standardized human pedigree nomenclature (8). Filled symbols represent affected individuals, open symbols denote unaffected family members. Diagonal slashes indicate deceased subjects. The short solid arrow adjacent to symbol IV-1 designates the proband. The pedigree demonstrates an autosomal-dominant inheritance pattern (Figure 2).

**Figure 2:**
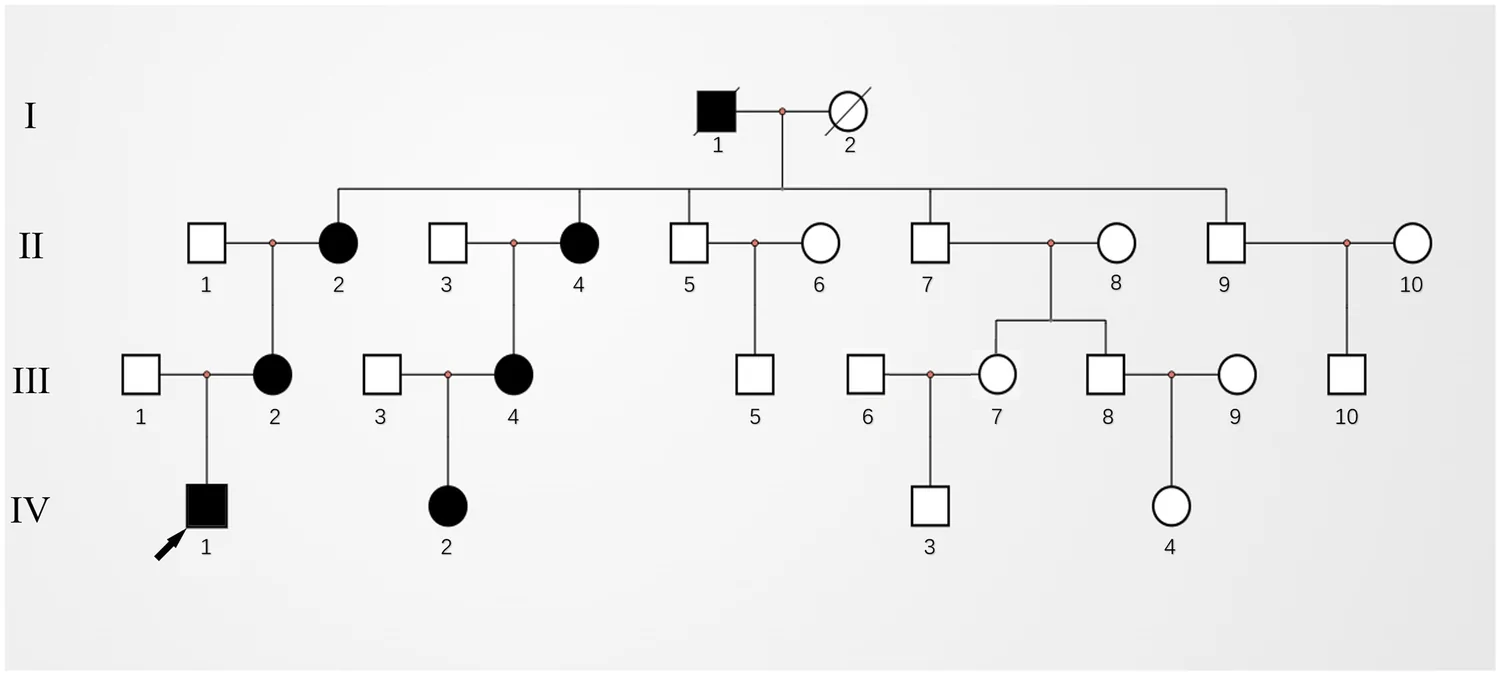
Pedigree chart of the family.

### 2.6 Auxiliary examinations

Pre-admission and post-admission laboratory findings are summarised in Table 2, with measured values and corresponding reference ranges provided. Imaging and specialist assessment results are described below.

**Table 2:** Summary of laboratory findings.

Parameter	Measured value	Reference range
Pre-admission investigations		
25-hydroxy vitamin D[25(OH)D]	20.692 ng/mL↓	30-100 ng/mL
Alkaline phosphatase	420 U/L↑	45-125 U/L
24-h urine calcium	4.12 mmol/24h	2.5-7.5 mmol/24h
24-h urine phosphorus	19.68 mmol/24h	12-42 mmol/24h
Parathyroid hormone (PTH)	17.23 pg/mL	15-65 pg/mL
Osteocalcin	11.30 ng/mL	10-40 ng/mL
*β*-CrossLaps (β-CTX)	1.41 ng/mL↑	0.21-0.44 ng/mL
Procollagen type I N-terminal propeptide (PINP)	389.20 ng/mL↑	19-84 ng/mL
Thyroid-stimulating hormone (TSH)	2.411 uIU/mL	0.27-4.2 uIU/mL
Free triiodothyronine (FT3)	6.09 pmol/L	3.1-6.8 pmol/L
Free thyroxine (FT4)	18.67 pmol/L	12-22 pmol/L
Vitamin A	0.41 mg/mL	
Post-admission Laboratory Tests		
Bone Metabolism Markers		
Total Vitamin D	26.89 ng/mL↓	30-100 ng/mL
Osteocalcin	145.50 ng/mL ↑	10-40 ng/mL
PINP	543.70 ng/mL↑	19-84 ng/mL
Bone-specific alkaline phosphatase (BALP)	>117.00 μg/L↑	<21.3 μg/L
24-h urine calcium	4.75 mmol/24h	2.5-7.5 mmol/24h
24-h urine inorganic phosphorus	20.62 mmol/24h	12-42 mmol/24h
Serum calcium	2.45 mmol/L	2.11-2.52 mmol/L
Serum inorganic phosphorus	1.79 mmol/L	1.29-1.94 mmol/L
PTH	21.8 pg/mL	15-65 pg/mL
Pituitary-target-gland hormones		
Luteinizing hormone (LH)	3.17 mIU/mL	1.2-8.6 mIU/mL
Follicle-stimulating hormone (FSH)	5.01 mIU/mL	1.3-10.1 mIU/mL
Testosterone	8.55 nmol/L	1.6-8.8 nmol/L
Progesterone	1.430 nmol/L↑	<1.27 nmol/L
Estradiol	46.13 pmol/L	<110 pmol/L
Prolactin (PRL)	23.20 ng/mL↑	4.1-18.4 ng/mL
Repeat PRL	6.04 ng/mL	4.1-18.4 ng/mL
Growth hormone (GH)	1.00 ng/mL	<5 ng/mL
Insulin-like growth factor-I (IGF-I)	453 ng/mL↑	126-414 ng/mL
Repeat IGF-I	427 ng/mL↑	126-414 ng/mL
Adrenocorticotropic hormone (ACTH)	16.40 pg/mL	7.2-63.3 pg/mL
Cortisol (morning)	7.97 μg/dL	4.0-22.3 μg/dL
Other laboratory tests		
Serum IgE	575.00 IU/mL↑	<150 IU/mL
Anti-thyroid peroxidase antibody (TPOAb)	46.03 U/mL↑	<34 U/mL
Anti-thyroglobulin antibody (TgAb)	<1.30 IU/mL	<4.11 IU/mL

CBC and trace-element testing (copper, iron, calcium, magnesium, lead, zinc) were performed in an external hospital and showed unremarkable findings. Routine urinalysis, routine stool examination, electrolytes, liver and kidney function, blood glucose, lipids, myocardial enzyme profiles and antinuclear antibody profile were tested at our hospital and within normal limits. All above-mentioned normal results are not listed in the table. ↑ = above reference range; ↓ = below reference range.

### 2.7 Imaging and other special examinations

- Bone Densitometry: Bone age and regional-site bone mineral density (Lumbar spine L1-4, femoral neck, total hip) were assessed at an external hospital. Bone age was 12 years and 10 months. BMD Z-scores from the external-hospital scan were as follows: Lumbar spine L1-4: −1.7; Femoral neck: −2.7; Total hip: −2.3. After admission, a total-body-less-head (TBLH) DXA scan was performed at our institution, yielding a Z-score of −2.2（BMD 0.614 g/cm^2^）, consistent with low-for-age bone mass.
- **x-ray:** Posteroanterior chest DR showed no definite abnormalities. DR of the bilateral upper extremities and full-length lower limbs revealed reduced bone radiodensity. DR of the thoracolumbar spine (AP & lateral), skull (AP & lateral), and pelvis (AP) demonstrated: ① Leftward curvature of the spine with kyphosis; ② Diffuse reduction in bone radiodensity; ③ Mild flattening of the T8 and T9 vertebral bodies, suggestive of possible low-trauma microfractures. Evidence of low-for-age bone mass together with the patient's history of recurrent fragility fractures supported the presence of impaired bone strength.
- **Magnetic Resonance Imaging (MRI):** Pituitary gland plain scan showed a slightly full pituitary gland(The mild elevation of IGF-1 levels is most likely related to physiological changes of puberty. Given the slightly increased IGF-1 and prolactin levels noted on initial testing, pituitary magnetic resonance imaging (MRI) was performed to exclude structural lesions. No pituitary lesions were found, with no impact on clinical management.
- **Ultrasound:** Abdominal (liver, gallbladder, pancreas, spleen), cardiac structure and function, bilateral testes and epididymis, kidneys, ureters, bladder, prostate, thyroid, and cervical lymph nodes showed no significant abnormalities.
- **Electrocardiogram (ECG):** Sinus arrhythmia, left axis deviation, otherwise within normal limits.
- **Specialist Consultations:** Dental evaluation showed no significant abnormalities in dentition or tooth structure; panoramic tomography indicated normal jawbone development. Otorhinolaryngology hearing examination and pure-tone audiometry were unremarkable.

### 2.8 Genetic testing

Genetic testing was performed using targeted-panel sequencing rather than whole-exome sequencing. The custom panel specifically covered *COL1A1* and *COL1A2*, because pathogenic variants within these two genes account for approximately 90% of OI cases, making them the primary focus for molecular diagnosis in patients with typical clinical phenotypes. Genetic testing was performed for the proband and his parents. Variant nomenclature was formally validated using VariantValidator (9). The proband carries a heterozygous frameshift *COL1A1* variant; his mother is heterozygous for the identical variant, whereas his father is wild-type. According to ACMG criteria, this variant is classified as Likely Pathogenic and predicted to cause *COL1A1* haploinsufficiency due to premature translation termination. Complete phenotypic co-segregation of this variant is observed across the multigenerational pedigree. Full variant details including HGVS nomenclature, genomic coordinates and zygosity are summarized in Table 3. To our knowledge, this exact c.358dup variant has not been previously reported in public variant databases or published osteogenesis imperfecta-related literature, although other truncating variants at codon 120 of *COL1A1* are known to be associated with mild osteogenesis imperfecta phenotypes. Consistent with modern OI classification recommendations, we refrain from directly mapping this genetic variant to one of the four historical Sillence subtypes. Nevertheless, the proband and affected family members exhibit clinical phenotypes consistent with OI type 1, and a clinical diagnosis of OI type 1 remains appropriate for this pedigree.

**Table 3:** Genetic testing results of the patient and his parents.

Gene	Variant Position	HGVS	Variant Type	Zygosity	Variant classification
*COL1A1*	NC_000017.11:g.50199433dup	NM_000088.4:c.358dup p.(Arg120ProfsTer49)	Frameshift variant	Proband:Heterozygous Father:Wild-type Mother:Heterozygous	Likely pathogenic

Public variant databases including gnomAD (v4.1.2), LOVD-*COL1A1* (LOVD platform v3.0), ClinVar (accessed Sep 7, 2026), and ClinGen (accessed Sep 7, 2026) were queried to evaluate the novelty of NM_000088.4:c.358dup. No matching variant entries were retrieved from all above-mentioned databases. No corresponding allele-frequency entry for this variant was present in gnomAD. ACMG/AMP 2015 criteria were applied for variant classification.

### 2.9 Diagnosis

Combining the patient's clinical manifestations (blue sclerae since birth, multiple low-trauma fractures, low BMD), family history (features consistent with autosomal dominant inheritance), and genetic test results (pathogenic heterozygous variant in the *COL1A1* gene), a definitive diagnosis of OI type1 was established.

### 2.10 Patient management and follow-up outcomes

After establishing the diagnosis, comprehensive anti-osteoporotic and supportive treatment was initiated, including calcium and active vitamin D supplementation, sequential anti-resorptive therapy with denosumab followed by zoledronic acid, as well as rehabilitation guidance. No adverse drug events occurred during anti-resorptive treatment. The patient completed 6-months of clinical follow-up. No new fragility fractures were observed, and pre-existing joint pain was substantially relieved. Detailed dosing, duration and rationale for this therapeutic switch are described in the Discussion section.

## 3 Discussion

### 3.1 Management of the present patient

Currently, there is no curative treatment for OI. The treatment principles aim to increase BMD, reduce fracture risk, improve skeletal deformities, and enhance quality of life. A comprehensive treatment approach was adopted for this patient:

1. **Basic Therapy:** Oral elemental calcium 600 mg once daily and calcitriol 0.25 μg every other day were administered for calcium and active vitamin D supplementation. Since infancy, the patient had only received intermittent calcium and vitamin-D supplements, rather than consistent adequate intake. Laboratory testing demonstrated reduced serum 25-hydroxyvitamin-D (20.692 ng/mL), accompanied by low bone mass and a history of recurrent fragility fractures. Therefore, calcium and calcitriol were prescribed. Calcitriol was given to enhance intestinal calcium absorption and optimize bone mineralization, which constitutes a specific clinical indication for this pediatric OI patient at high fracture risk.
2. **Anti-osteoporotic Pharmacotherapy:** Subcutaneous denosumab 60 mg was administered once. Afterwards, intravenous zoledronic acid 5 mg was given every 12 months for long term maintenance. This regimen aims to inhibit osteoclast activity, reduce bone resorption, and increase BMD.The switch from denosumab to zoledronic acid followed a pre defined sequential anti resorptive protocol for pediatric OI at our center. Denosumab was chosen for rapid initial suppression of high bone turnover. Zoledronic acid was subsequently introduced for sustained long term bone protection, to mitigate the risk of rebound hypercalcemia associated with repeated denosumab exposure in children. This therapeutic change was not triggered by drug related adverse effects, inadequate clinical response, or limited drug availability. For this 13-year-old pubertal male patient weighing 55 kg, intravenous zoledronic acid 5 mg was administered once-yearly. This fixed-dose regimen is suitable for patients with a body-weight above 25 kg and has been reported in relevant therapeutic studies (10). The patient tolerated treatment well during follow-up, with no hypocalcaemia or other bisphosphonate-related adverse events.
3. **Rehabilitation and Lifestyle Guidance:** Avoid strenuous exercise and trauma to prevent fractures. Engage in appropriate rehabilitation exercises such as swimming and walking to enhance muscle strength and protect bones. Regularly monitor BMD, bone metabolism markers, and liver/kidney function to adjust the treatment plan accordingly.
4. **Treatment outcome:** The patient tolerated the treatment well with no adverse drug reactions. He was followed up for 6 months. During follow up, no new fractures occurred and joint pain was relieved. BMD was improved compared with baseline. The patient returned to normal school life with significantly improved quality of life.

### 3.2 Advances in OI therapy

Current treatment for OI is comprehensive, including basic therapy, pharmacotherapy, surgical intervention, and rehabilitation (1). Basic therapy primarily involves calcium and vitamin D supplementation to maintain normal serum calcium levels and provide substrates for bone mineralization. Pharmacotherapy mainly consists of bisphosphonates, such as zoledronic acid and pamidronate disodium. These drugs inhibit osteoclast activity, reduce bone resorption, increase BMD, and decrease fracture incidence, and are currently first-line agents for OI treatment (11). Denosumab, a monoclonal antibody that specifically binds to receptor activator of nuclear factor-*κ*B ligand (RANKL), inhibits osteoclast formation and activity and has recently been used in OI treatment. It has shown non-inferiority to alendronate in improving bone endosteal disease and may enhance vertebral remodeling and height gain in pediatric patients. However, its use requires risk stratification due to phenomena like rebound hypercalcemia in children and its efficacy being hormone-dependent in adults; therefore, continuous monitoring of serum calcium levels and careful calcium supplementation during treatment are essential (12, 13). Denosumab has the characteristics of rapid onset and strong targeted antiresorptive effect, which can quickly control the progression of bone lesions in the early stage; zoledronic acid, as a long-acting bisphosphonate, can sustainably inhibit osteoclast activity, stabilize bone mineral density. Rebound hypercalcemia is a known side effect after denosumab therapy and is more commonly reported in pediatric patients. Studies have shown that bisphosphonates can be used for the treatment of rebound hypercalcemia in pediatric patients induced by denosumab. The sequential application of these two therapies is likely to provide more clinical benefits for patients with immature skeletal development (14). Teriparatide, a human 1−34 parathyroid hormone, has osteoanabolic and fracture protective effects in patients with osteoporosis. Teriparatide therapy has been reported to improve bone mineral density and parameters of vertebral bone strength in adults with type 1 OI. Currently, due to the potential risk of osteosarcoma in adolescents with unfused epiphyses and the lack of clinical evidence regarding its safety and efficacy in pediatric populations, teriparatide is universally contraindicated in children under 18 years of age, adolescents with open growth plates, and pediatric OI (15, 16). Surgical intervention is primarily indicated for correcting severe, progressive long bone bowing deformities, stabilizing recurrent pathological fractures, and managing severe scoliosis. Concurrently, systematic rehabilitation therapy is central to lifelong management. Its goals are to enhance muscle strength and endurance, improve joint mobility and motor function, thereby maximizing the patient's activity level, independence, and quality of life, and indirectly contributing to fracture risk reduction. Furthermore, gene therapy is a research direction for OI treatment, currently in preclinical or early clinical trial stages, and holds promise for providing a curative approach for OI in the future (17, 18).

### 3.3 Correlation between *COL1A1* gene variants and OI

The *COL1A1* gene is located at 17q21.33, spans approximately 18 kb, contains 51 exons, and encodes the *α*1 chain of type I collagen. Type I collagen is a major component of connective tissues such as bone, skin, tendons, and ligaments. Variants in the *COL1A1* gene can lead to reduced synthesis or structural abnormalities of type I collagen, diminishing the mechanical strength of bone and resulting in OI (1). Variant types in the *COL1A1* gene are diverse, including missense, frameshift, nonsense, and splice site variants. Different variant types are closely associated with the clinical phenotype of OI (7, 18) Frameshift variants often lead to reduced collagen synthesis and are commonly associated with OI type 1. Missense variants, particularly those affecting the triple helical structure of collagen, may result in more severe phenotypes such as OI types 2 or 3 (19, 20). The variant identified in this patient, a heterozygous frameshift variant in the *COL1A1* gene, p.(Arg120ProfsTer49), causes a frameshift in the amino acid sequence and introduces a premature termination codon. This likely results in reduced synthesis of the pro-α1(I) collagen chain, consistent with the patient's milder clinical phenotype (OI type 1). This finding aligns with the previously established correlation between *COL1A1* variant types and OI phenotypes reported in classic literature (19, 21). Large cohort analyses have consistently shown that *COL1A1* haploinsufficiency variants are associated with the mildest form of OI, type 1, characterized by recurrent low-trauma fractures, congenital blue sclerae, normal or near-normal stature, and absence of severe skeletal deformities. Similar phenotypes have been reported in other patients with functionally equivalent frameshift variants in the N-terminal region of *COL1A1*, further supporting the genotype–phenotype correlation observed in our case (22). We queried multiple publicly available variant databases, including ClinVar and LOVD-OI (Leiden Open Variation Database for osteogenesis imperfecta), for the exact frameshift variant NM_000088.4:c.358dup [p.(Arg120ProfsTer49)]. No entries corresponding to this precise duplication variant were retrieved. To our knowledge, this c.358dup variant has not been previously reported in the published literature or in clinical variant repositories. Although a small number of other pathogenic truncating variants affecting codon 120 of *COL1A1* have been documented in patients with mild OI (type 1) phenotypes, the number of such previously reported cases remains limited, and none carries the identical duplication identified in our patient. Consistent with its autosomal-dominant inheritance pattern, this variant showed complete co-segregation with the OI type 1 phenotype across the multigenerational pedigree, and the proband's mother, who carries the same heterozygous variant, also exhibited typical clinical manifestations of OI type 1. The absence of prior reports of this precise variant, together with the consistent mild phenotype and full family co-segregation, underscore both the novelty and the clinical relevance of this finding.

### 3.4 Novel aspects of the present case

This multigenerational pedigree-based case report defines its main contributions to existing osteogenesis imperfecta literature in two key respects. First, we report a previously undescribed *COL1A1* frameshift variant NM_000088.4:c.358dup p.(Arg120ProfsTer49). Complete phenotype-co-segregation across the pedigree supports its pathogenic role for OI type 1, adding another variant-phenotype correlation for *COL1A1-*related mild osteogenesis imperfecta. Second, we present 6-month clinical follow-up data describing the therapeutic response of an adolescent OI type 1 patient treated with sequential denosumab-to-zoledronic-acid anti-resorptive therapy. This adds practical observational evidence for this sequential regimen in paediatric OI management, which remains sparsely documented in published work.

## 4 Conclusion

This 13-year-old male patient, presenting with blue sclerae and multiple low-trauma fractures, was definitively diagnosed with OI type 1 caused by a heterozygous *COL1A1* gene variant [NM_000088.4:c.358dup p.(Arg120ProfsTer49)], based on family history, imaging findings, and genetic testing. The diagnosis of OI necessitates a comprehensive evaluation integrating clinical manifestations, family history, imaging, and genetic testing results. Genetic testing can identify the causative gene and confirm the subtype, providing a basis for clinical management and genetic counseling. Current treatment for OI is primarily comprehensive, with bisphosphonates and denosumab being commonly used anti-osteoporotic agents that can effectively increase BMD and reduce fracture risk. Future efforts should focus on advancing genetic research in OI to promote the development of gene therapy, aiming to provide more effective treatment options for OI patients. Future research could further explore the profiles of bone metabolism markers, imaging characteristics, and optimization of intervention strategies for pediatric OI.

## Data Availability

The datasets presented in this article are not readily available because of ethical and privacy restrictions. Requests to access the datasets should be directed to the corresponding authors.

## References

[1] Marini JC Forlino A Bächinger HP Bishop NJ Byers PH Paepe AD. Osteogenesis imperfecta. Nat Rev Dis Primers. (2017) 3:17052. 10.1038/nrdp.2017.52 28820180

[2] Hasegawa K. Osteogenesis imperfecta: pathogenesis, classification, and treatment. Clin Pediatr Endocrinol. (2025) 34(3):152–61. 10.1297/cpe.2025-0009 40636817 PMC12236168

[3] Sillence DO Senn A Danks DM. Genetic heterogeneity in osteogenesis imperfecta. J Med Genet. (1979) 16(2):101–16. 10.1136/jmg.16.2.101 458828 PMC1012733

[4] Van Dijk FS Sillence DO. Osteogenesis imperfecta: clinical diagnosis, nomenclature and severity assessment. Am J Med Genet A. (2014) 164(6):1470–81. 10.1002/ajmg.a.36545 Erratum in: Am J Med Genet A. (2015) 167A(5):1178. PMC4314691 24715559

[5] Sillence DO. A dyadic nosology for osteogenesis imperfecta and bone fragility syndromes 2024. Calcif Tissue Int. (2024) 115(6):873–90. 10.1007/s00223-024-01248-7 38942908 PMC11607092

[6] Sait H Adarsha N Moirangthem A Saxena D Sharma L Dabadgao P. Molecular and clinical landscape of osteogenesis imperfecta: unraveling autosomal recessive forms, therapeutic outcomes, and bone mineral density in carriers. Clin Genet. (2025) 108(6):684–95. 10.1111/cge.70003 40650436

[7] Chinese Society of Osteoporosis and Bone Mineral Research. Guidelines for the diagnosis and treatment of osteogenesis imperfecta (2022 version). Chin J Osteoporos Bone Miner Res. (2022) 15(5):441–60. 10.12114/j.issn.1007-9572.2023.0121

[8] Bennett RL French KS Resta RG Austin J. Practice resource-focused revision: standardized pedigree nomenclature update centered on sex and gender inclusivity: a practice resource of the national society of genetic counselors. J Genet Couns. (2022) 31:1238–48. 10.1002/jgc4.1621 36106433

[9] Freeman PJ Hart RK Gretton LJ Brookes AJ Dalgleish R. Variantvalidator: accurate validation, mapping and formatting of sequence variation descriptions. Hum Mutat. (2018) 39(1):61–8. 10.1002/humu.23348 28967166 PMC5765404

[10] Sun L Hu J Liu J Zhang Q Wang O Jiang Y. Relationship of pathogenic mutations and responses to zoledronic acid in a cohort of osteogenesis imperfecta children. J Clin Endocrinol Metab. (2022) 107(9):2571–9. 10.1210/clinem/dgac366 35727737

[11] Ying ZM Hu B Yan SG. Oral bisphosphonate therapy for osteogenesis imperfecta: a systematic review and meta-analysis of six randomized placebo-controlled trials. Orthop Surg. (2020) 12(4):1293–303. 10.1111/os.12611 32589343 PMC7454147

[12] Stasek S Reincke S Rehberg M Semler O Hoyer-Kuhn H. Urinary calcium and bone resorption markers during 3 years of denosumab treatment in pediatric osteogenesis imperfecta. JBMR Plus. (2025) 9(10):ziaf140. 10.1093/jbmrpl/ziaf140 40978125 PMC12448440

[13] Mei Y Cai S Jiang Y Tian Y Shen L Gu J. Denosumab in patients with osteogenesis imperfecta and a historical control study with alendronate. Front Endocrinol (Lausanne). (2025) 16:1445093. 10.3389/fendo.2025.1445093 40496553 PMC12148890

[14] Gandolfi A Shaaban S. Denosumab-induced rebound hypercalcemia treated with bisphosphonates in a pediatric patient. JCEM Case Rep. (2023) 1(5):luad133. 10.1210/jcemcr/luad133 37908209 PMC10612474

[15] Chinese Association for Rehabilitation Technology Transformation and Development, Bone and Joint Group of Chinese Society of Osteoporosis and Bone Mineral Research, Osteoporosis Working Committee of Chinese Medical Doctor Association Orthopedics Branch. [Chinese expert consensus on treatment of osteoporotic fractures with teriparatide (2024 edition)]. Zhonghua yi xue za zhi. (2024) 104(17):1456–65. 10.3760/cma.j.cn112137-20231101-00964 38706051

[16] Liu W Nicol L Orwoll E. Current and developing pharmacologic agents for improving skeletal health in adults with osteogenesis imperfecta. Calcif Tissue Int. (2024) 115(6):805–11. 10.1007/s00223-024-01188-2 38472351

[17] Muldiiarov V Buesing K Wallace MJ. Pediatric scoliosis in osteogenesis imperfecta: from genetic mechanisms to therapeutic strategies. Orthop Surg. (2025) 17(11):3036–47. 10.1111/os.70170 40988516 PMC12580250

[18] Chaugule S Constantinou CK John AA Micha D Eekhoff M Gravallese E. Comprehensive review of osteogenesis imperfecta: current treatments and future innovations. Hum Gene Ther. (2025) 36(5–6):597–617. 10.1089/hum.2024.191 39932815 PMC11971546

[19] Deng H Huang X Yuan L. Molecular genetics of the COL2A1-related disorders. Mutat Res Rev Mutat Res. (2016) 768:1–13. 10.1016/j.mrrev.2016.02.003 27234559

[20] Beck K Chan VC Shenoy N Kirkpatrick A Ramshaw JAM Brodsky B. Destabilization of osteogenesis imperfecta collagen-like model peptides correlates with the identity of the residue replacing glycine. Proc Natl Acad Sci U S A. (2000) 97(8):4273–8. 10.1073/pnas.070050097 10725403 PMC18226

[21] Willing MC Cohn DH Byers PH. Frameshift mutation near the 3’ end of the *COL1A1* gene of type I collagen predicts an elongated pro alpha 1(I) chain and results in osteogenesis imperfecta type I. J Clin Invest. (1990) 85(1):282–90. 10.1172/JCI114424 2295701 PMC296416

[22] Li L-J Lyu F Song Y-W Wang O Jiang Y Xia W-B. Genotype-phenotype relationship in a large cohort of osteogenesis imperfecta patients with *COL1A1* mutations revealed by a new scoring system. Chin Med J (Engl). (2019) 132(2):145–53. 10.1097/CM9.0000000000000013 30614853 PMC6365277

